# Real-time comparative evaluation of bioMerieux VITEK MS versus Bruker Microflex MS, two matrix-assisted laser desorption-ionization time-of-flight mass spectrometry systems, for identification of clinically significant bacteria

**DOI:** 10.1186/s12866-014-0289-0

**Published:** 2014-11-30

**Authors:** Wafaa Jamal, M John Albert, Vincent O Rotimi

**Affiliations:** Department of Microbiology, Faculty of Medicine, Kuwait University and Microbiology Unit, Mubarak Al-Kabir Hospital, Jabriya, Kuwait; Department of Microbiology, Faculty of Medicine, Kuwait University, P.O. Box 24923, Safat, 13110 Kuwait

**Keywords:** Evaluation, MALDI-TOF mass spectrometry, Clinical bacterial isolates

## Abstract

**Background:**

Matrix-assisted laser desorption-ionization time-of-flight mass spectrometry (MALDI-TOF MS) recently became available for the identification of bacteria in routine diagnostic laboratories. It is rapid and cost-effective and likely to replace phenotypic identification. This study was undertaken to compare two MALDI-TOF MS-based, Bruker Microflex MS (BMS) and VITEK MS (VMS) systems, for identification (ID) of clinically significant bacterial isolates. Clinically relevant broad diversity of bacterial isolates obtained during a 6-consecutive months of routine laboratory processing of clinical specimens were subjected to ID by the BMS and VMS in parallel with Vitek 2, a conventional phenotypic system (CPS). For the BMS, the isolates were tested in duplicates directly and after pretreatment. Identification was provided with accompanying scores according to manufacturers’ instructions. With VMS, single deposits of the same sets of isolates were tested in duplicates directly on MALDI-plate. Results were interpreted according to the manufacturer’s protocols. Discrepant results were resolved by 16S rRNA gene amplification and sequencing.

**Results:**

A total of 806 pathogens comprising 507 Gram-negative bacilli (GNB), 16 Gram-negative cocci (GNC), 267 Gram-positive cocci (GPC), and 16 Gram-positive bacilli (GPB) were tested. BMS and VMS correctly identified isolates to genus and species levels (ID 97.3% and 93.2%, and 99.8% and 99.0%, respectively). Both systems as well as the CPS correctly identified the majority of the species in the family *Enterobacteriaceae*, *Pseudomonas* spp., and *Acinetobacter baumannii*. Turnaround time for identification by BMS and VMS was <20 min compared with 24-48 h by the CPS.

**Conclusions:**

VMS performed slightly better than BMS with GPC ID, especially the *Streptococcus* spp. Some *S. mitis* isolates were identified as *S. pneumoniae* by BMS. BMS and VMS were rapid and proved to be consistently accurate for producing bacterial identification in a fraction of time it takes for identification by CPS.

## Background

The routine identification of bacterial isolates in the hospital clinical diagnostic microbiology laboratory is currently done by analysis of phenotypic characteristics such as growth on selective and non-selective media, colonial morphology, Gram-stain morphology and typical biochemical reactions. These methods are laborious, time-consuming and require a relatively long turn-around time. Fast and cost-effective matrix-assisted laser desorption ionization-time of flight mass spectrometry (MALDI-TOF MS)-based systems have been developed essentially to replace the biochemical and other phenotypic systems for routine identification of bacteria. This is because it is now necessary to have a low-cost, fast and reproducible method for bacterial identification in the routine diagnostic laboratory.

Initially, MALDI-TOF MS technique was developed for industrial use to analyze and identify proteins. However, two decades later, Holland *et al*., [1] and Krishnamurthy and Ross [2] demonstrated that it could be used for rapid identification of bacterial whole–cell based on spectral pattern. But its potential for routine use in microbial identification in clinical microbiology laboratory was only demonstrated a few years ago [3-6]. This technique is based on the analysis of microbial proteins by an ionization process devoid of fragmentation by coordinated action of laser and small organic acids of the matrix. These are then separated on the basis of their mass-to-charge ratios, a process which results in a characteristic mass spectral profile. Identification of microbes is based on the comparison of the protein spectrum generated from intact whole microbial cells to a database of species-specific reference protein profiles. Protein analysis by MALDI-TOF MS does not require lengthy biochemical reactions, thereby making it a more rapid identification strategy than traditional routine methods [5,6].

Currently, commercial and user-friendly instruments containing different algorithms for the identification of microbial protein mass spectrum pattern along with a database for thousands of reference bacteria are now available. These are Bruker Microflex MS (Bruker Daltonics, Bremen, Germany), VITEK MS IBB (bioMerieux, Marcy l’Etoile, France), VITEK MS RUO (formerly Saramis, bioMerieux) and Andromas system (Andromas SAS, Paris, France) [7,8]. Many laboratories have focused on analysis of specific bacterial genera using the MALDI-TOF MS technology [9-14]. Other studies have analyzed general groups of bacteria [15,16], and yeasts [17,18], or evaluated its use in the routine clinical laboratory for bacterial identification [3-6,19,20].

In the current study, we carried out a comparative evaluation of the performance of Bruker Microflex MS (BMS, Daltonics) versus VITEK MS (VMS, bioMerieux) based on an updated classifier and database, in parallel with routine phenotypic Vitek 2 system (CPS, bioMerieux) and molecular methods i.e. 16S rRNA amplification and sequencing.

## Methods

### Bacterial isolates

Consecutive, clinically relevant, a broad diversity of bacterial isolates were tested during a period of six consecutive months (January 2012 to June 2012) of routine laboratory processing of clinical specimens in the Microbiology Laboratory, Mubarak Al Kabir hospital, Kuwait. Duplicate isolates from the same patient were excluded. The 806 isolates included in the study encompassed *Enterobacteriaceae*, non-fermentative Gram-negative bacilli, other Gram-negative bacteria, staphylococci and related species, streptococci and related species, and Gram-positive rods. They were isolated from different sources- blood, urine, pus, tissue, wound, respiratory secretion and body fluid. All identification tests were carried out simultaneously by BMS, VMS and CPS as a part of routine diagnostic workup.

### Vitek 2 system

Routine bacterial identification in the laboratory was carried out by Vitek 2 (bioMerieux). Gram-positive and Gram-negative isolates were inoculated into GP ID (card no. 21342) and GN ID (card no. 21341) respectively. *Neisseria* and *Haemophilus* species were identified by NH ID (card no. 21346). When necessary, complementary biochemical tests were done.

### MALDI-TOF Bruker MS (BMS)

Measurements were done with a Microflex LT mass spectrometer (Bruker Daltonics) using FlexControl software (version 3.3). The spectra were imported into the integrated MALDI Biotyper software (version 3.0) and analyzed by standard pattern matching with a default setting. The isolates were tested in duplicates directly and after pretreatment. A colony from blood agar plate was directly spotted on the MALDI plate, and then overlaid with 1 μl of matrix solution and air-dried. The loaded plate was then placed in the instrument according to the manufacturer’s instructions. The spectrum of each isolate was compared with that in the database and the identification was provided by a score of reliability (<1.7 = ID not reliable; ≥1.7 and <2.0 = ID at genus level; ≥2.0 = ID at species level). A result was considered species consistent if all matches with a log score ≥2.0 were of the same species and if matches with a log score between 2.0 and 1.7 correspond only to other species of the same genus. A genus consistent result was accepted if all log scores ≥1.7 belong to the same genus.

### MALDI TOF VITEK MS (VMS)

The isolates were tested in duplicates directly on the MALDI-plate. A single bacterial colony was deposited on the target slide, followed by the addition of the matrix (VITEK MS-CHCA) and air drying. The loaded slide was inserted into the VITEK MS machine. VITEK mass spectrometer was used to generate spectra of the bacterial suspension and the Biotyper software (version 2.0) was used to analyze the results. Microbial identification was achieved by obtaining the spectra using MALDI-TOF technology and analyzing the spectra with the VITEK MS database. The peaks from these spectra were compared with the characteristic pattern for the species, genus or family of the microorganism, leading to identification of the organism. The results were evaluated according to a colored index: green for ≥90% identity, yellow for 85–89.9% identity and white for <85% identity. All of the identifications to the genus or species level fell into the green zone, with a score of >90% considered reliable. Scores between 85 and 90% were considered as acceptable identification. A cut-off of 90% was chosen for the VITEK MS.

### Quality control

*Escherichia coli* ATCC 8739 was included as a positive control and matrix alone with no organism was included as a negative control in each run in both MALDI-TOF systems.

### Discrepancy

Discrepancies were defined as different genus- or species-level identification obtained from the VITEK MS, Microflex Bruker MS or conventional method Vitek 2. Whenever there was a discrepancy, the analysis was repeated by both systems to eliminate the possibility of contamination. Thereafter, any other disagreements in the ID were resolved by performing 16S rRNA gene amplification and sequencing on the discrepant isolates.

### 16S rRNA gene amplification

DNA from colonies obtained from samples with discordant results emanating from the 3 identification systems was extracted as previously described by Boom *et al.* [21]. The 16S rRNA genes were amplified and sequenced using universal 16S rRNA-specific primers [22]. The sequences obtained were compared with sequences present in the GeneBank database using BLAST software (http://www.ncbi.nih.gov).

### Ethics statement

The evaluation of diagnostic systems was performed in the routine Clinical Microbiology Laboratory whose job is to provide support for patient care. Ethical approval is not required for analysis of routine clinical specimens sent to this laboratory.

## Results

A total of 806 clinically significant pathogens comprising 507 Gram-negative bacilli (GNB), 16 Gram-negative cocci (GNC), 267 Gram-positive cocci (GPC), and 16 Gram-positive bacilli (GPB) made up of 39 genera and 70 species were tested during the study period, as shown in Tables 1, 2, 3 and 4. Of the 806 isolates, conventional Vitek 2 system correctly identified 795 (98.6%) to the genus level and 777 (96.4%) to the species level. VITEK MS identified 805 (99.9%) to the genus level and 798 (99.0%) to the species level. Bruker Microflex MS identified 784 (97.3%) and 751 (93.2%) to the genus and species levels, respectively; 17 isolates were also correctly identified to the species level but at no reliable ID score of <1.7. Both MALDI-TOF systems took less than 20 minutes from colony to ID result compared with ≥24 h by CPS.

**Table 1:** **Discrepancies and error in the conventional identification method (Vitek 2) and MALDI-TOF for the identification of 507 Gram-negative bacteria**

**Final identification (no of isolates)**	**No of isolates**						
	**Conventional identification method (Vitek 2)**		**VITEK MS identification**		**Bruker MS identification**		**16S rRNA sequence result (number of discrepant organisms sequenced)**
	**No ID**	**Mis-ID**	**No ID**	**Mis - ID**	**No ID**	**Mis-ID**	
*Escherichia coli* (181)	0	0	0	0	0	0	
*Klebsiella pneumoniae* (71)	0	0	0	0	0	1 (score <1.7)	*K. pneumoniae* (1)
*Klebsiella oxytoca* (2)	0	0	0	0	0	0	
*Pseudomonas aeruginosa* (66)	0	0	0	0	0	0	
*Acinetobacter baumannii* (39)	0	0	0	0	0	1 (*Acinetobacter* genomospecies 13TU)*, 3 (score <1.7)	*A. baumannii* (4)
*Acinetobacter lwoffii* (2)	0	0	0	0	0	1 (score <1.7)	*A. lwoffii* (1)
*Salmonella* spp. (29)	0	0	0	0	0	1 (score <1.7)	*Salmonella* spp. (1)
*Enterobacter cloacae* (14)	0	0	0	0	0	0	
*Enterobacter aerogenes* (7)	0	0	0	0	0	0	
*Enterobacter asburiae* (5)	0	0	0	0	0	0	
*Pantoea agglomerans* (1)	0	0	0	0	0	0	
*Proteus mirabilis* (17)	0	0	0	0	0	0	
*Stenotrophomonas maltophilia* (18)	0	0	0	0	0	1 (score <1.7)	*S. maltophilia* (1)
*Serratia marcescens* (15)	0	0	0	0	0	0	
*Citrobacter koseri* (9)	0	0	0	0	0	0	
*Citrobacter freundii* (1)	0	0	0	0	0	0	
*Morganella morganii* (7)	0	0	0	0	0	0	
*Achromobacter xylosoxidans* (5)	0	0	0	0	0	2 (*Acinetobacter* spp.)	*A. xylosoxidans* (2)
*Providencia rettgeri* (4)	0	2 (*Providencia stuartii*)	0	2 (*P. stuartii*)	0	1 (score <1.7)	*P. rettgeri* (2)
*Burkholderia cepacia* (2)	0	0	0	0	0	1 (score <1.7)	*B. cepacia* (1)
*Sphingomonas paucimobilis* (2)	0	0	0	0	0	2 (score <1.7)	*S. paucimobilis* (2)
*Aeromonas hydrophila/caviae* (2)	0	0	0	0	0	2 (*Aeromonas jandaei)*	*A. hydrophila/caviae* (2)
*Ralstonia solanacearum* (1)	0	1(*Ralstonia pickettii*)	0	1 (*R. pickettii*)	0	1 (*Ralstonia insidiosa*)	*R. solanacearum* (1)
*Chryseobacterium indologenes* (1)	0	0	0	0	0	0	
*Plesiomonas shigelloides* (1)	0	0	0	0	0	0	
*Vibrio parahaemolyticus* (1)	0	0	0	0	0	0	
*Campylobacter jejuni* (1)	0	0	0	0	0	0	
*Bordetella pertussis* (1)	0	0	0	0	0	0	
*Bordetella parapertussis* (1)	0	0	0	0	0	0	
*Yersinia enterocolitica* (1)	0	0	0	0	0	0	

No ID = no identification in spite of its presence in database; Mis-ID = misidentification; *Current name is *Acinetobacter nosocomialis.*

**Table 2:** **Discrepancies and error in the conventional identification method (Vitek 2) and MALDI-TOF for the identification of 267 Gram-positive bacteria**

**Final identification (no of isolates)**	**Number of isolates**						
	**Conventional identification method (Vitek 2)**		**VITEK MS identification**		**Bruker MS identification**		**16S rRNA sequence results (number of discrepant organism sequenced)**
	**No ID**	**Mis-ID**	**No ID**	**Mis-ID**	**No ID**	**Mis-ID**	
*Staphylococcus aureus* (53)	0	0	0	0	0	0	
*Staphylococcus epidermidis* (34)	0	1 (*Staphylococcus capitis*)	0	1 (*S. capitis*)	0	0	*S. epidermidis* (2)
*Staphylococcus haemolyticus* (11)	0	0	0	0	0	0	
*S. capitis* (11)	0	0	0	0	0	0	
*Staphylococcus hominis* (7)	0	0	0	0	0	0	
*Staphylococcus lugdunensis* (4)	0	0	0	0	0	0	
*Staphylococcus caprae* (1)	0	0	0	0	0	1 (*Staphylococcus auricularis*)	*S. caprae* (1)
*Staphylococcus warneri* (2)	0	0	0	*S.auricularis*	0	1 (*S. epidermidis*)	*S. warneri* (1)
*Streptococcus agalactiae* (46)	0	0	0	1 (*Nocardia asteroides*)	0	1 (*Alloiococcus otitis*), 1 *(Lactobacillus sakei*)	*S. agalactiae* (3)
*Rothia mucilaginosa* (1)	0	0	0	0	0	0	
*Enterococcus faecalis* (29)	0	0	0	0	0	0	
*Enterococcus faecium* (6)	0	0	0	0	0	0	
*Enterococcus raffinosus* (3)	0	0	0	0	0	0	
*Enterococcus avium* (1)	0	0	0	0	0	0	
*Streptococcus pyogenes* (7)	0	0	0	0	0	0	
*Micrococcus luteus/lylae* (6)	0	2 (*Kocuria kristinae*)	0	0	0	3 (1 *Clostridium beijeriuckii,* 2 *Propionibacterium acnes*)	*M. luteus* (5)
*Rhodococcus equi* (1)	NA	NA	NA	NA	0	0	*R. equi* (1)
*Streptococcus parasanguis* (3)	0	0	0	0	0	1 (*Alloiococcus otitis*)	*S. parasanguis* (1)
*Streptococcus salivarius* (1)	0	0	0	0	0	0	
*Streptococcus lutetiensis* (1)	0	1 (*Streptococcus infantarius*)	0	1 (*Streptococcus infantarius*)	0	0	*S. lutetiensis* (1)
*Streptococcus dysagalactiae* (2)	0	0	0	0	0	0	
*Streptococcus mitis/oralis* (26)	0	*3* (*Streptococcus constellatus*), 1 (*Streptococcus peroris*), 1 (*Streptococcus pluranimalium*), 1 (*Streptococcus pentosaceus*),1 (*Streptococcus sanguis*), 1 (*Streptococcus thoraltensis*)	0	1 (*S. constellatus*)	0	18 (*Streptococcus pneumoniae*)	*S. mitis / oralis* (26)
*Streptococcus anginosus* (2)	0	0	0	0	0	0	
*Streptococcus gallolyticus* (1)	0	0	0	0	0	1 *(Propionibacterium acnes*)	*S. gallolyticus* (1)
*Granulicatella adiacens* (2)	0	2 (*S. sanguinis, S. thoraltensis*)	0	0	0	2 *(S. pneumoniae, Streptococcus oralis*)	*G. adiacens* (2)
*S. pneumoniae* (5)	0	0	0	0	0	0	
*Streptococcus mutans* (1)	0	0	0	0	0	1 (*P. acnes)*	*S. mutans* (1)

No ID = no identification in spite of its presence in database; Mis - ID = misidentification; NA = not present in database

**Table 3:** **Discrepancies and error in the conventional identification method (Vitek 2) and MALDI-TOF for the identification of 16 Gram-negative cocci**

**Final identification (no of isolates)**	**Number of isolates**						
	**Conventional identification method (Vitek 2)**		**VITEK MS identification**		**Bruker MS identification**		**16S rRNA sequence results (number of discrepant organisms sequenced)**
	**No ID**	**Mis - ID**	**No ID**	**Mis - ID**	**No ID**	**Mis - ID**	
*Haemophilus influenzae* (8)	0	0	0	0	0	2 (score <1.7)	*H. influenzae* (2)
*Neisseria cinerea* (1)	0	0	0	1 (*Neisseria subflava*)	0	1 (*Neisseria sicca*)	*N. cinerea* (1)
*Neisseria subflava* (5)	NA	NA	0	0	0	*Neisseria flava* (5)	*N. subflava* (5)
*Neisseria meningitidis* (1)	0	0	0	0	0	0	
*Moraxella catarrhalis* (1)	0	0	0	0	0	0	

No ID = no identification in spite of its presence in database; Mis - ID = misidentification; NA = not present in database.

**Table 4:** **Discrepancies and error in the conventional identification method (Vitek 2) and MALDI-TOF for the identification of 16 Gram-positive bacilli**

**Final identification (no of isolates)**	**Number of isolates**						
	**Conventional identification method (Vitek 2)**		**VITEK MS identification**		**Bruker MS identification**		**16S rRNA sequence results (number of discrepant organisms sequenced)**
	**No ID**	**Mis - ID**	**No ID**	**Mis - ID**	**No ID**	**Mis - ID**	
*Corynebacterium terpenotabidum* (1)	NA	NA	0	1 (*Clostridium septicum*)	0	1 (*Clostridium beijerinckii*)	*C. terpenotabidum* (1)
*Lactobacillus jensenii* (3)	NA	NA	0	0	0	0	*L. jensenii* (3)
*Corynebacterium striatum* (5)	NA	NA	0	0	0	0	*C. striatum* (5)
*Lactobacillus rhamnosus* (2)	NA	NA	0	0	0	0	*L. rhamnosus* (2)
*Erysipelothrix rhusiopathiae* (1)	0	0	0	0	0	0	
*Listeria monocytogenes* (1)	0	0	0	0	0	0	
*Listeria ivanovi* (2)	0	0	0	0	0	1 (*L. monocytogenes*)	*L. ivanovii* (1)
*Nocardia otitidiscaviarum* (1)	0	1 (*L. monocytogenes*)	0	1 (*L. monocytogenes*)	0	1 (*L. monocytogenes*)	*N. otitidiscaviarum* (1)

No ID = no identification spite of its presence in database; Mis - ID = misidentification; NA = not present in database.

As shown in Table 1, both VMS and Vitek 2 gave correct and acceptable identification for 504/507 (99.4%) Gram-negative bacilli. Using 16S rRNA sequencing results as the gold standard, two *Providencia rettgeri* were misidentified as *Providencia stuartii* and one *Ralstonia solanacearum* was identified as *Ralstonia pickettii.* BMS gave correct and an acceptable identification for 490/507 (96.6%) Gram-negative bacilli. It could identify 11 Gram-negative bacilli (1 *Klebsiella pneumoniae*, 3 *Acinetobacter baumannii*, 1 *Acinetobacter lwoffii*, 1 *Salmonella* species, 1 *Stenotrophomonas maltophilia*, 1 *P. rettgeri*, 1 *Burkholderia cepacia*, and 2 *Sphingomonas paucimobilis*) correctly but with an unacceptable ID score of <1.7 when compared to sequencing results. However, the system misidentified 4 Gram-negative bacilli with an acceptable high ID score >2.0 (1 *A. baumannii* as *Acinetobacter* genomospecies 13TU [this organism has been recently named as *Acinetobacter nosocomialis,*http://www.bacterio.net/], 2 *Aeromonas hydrophila/caviae* as *Aeromonas jandaei*, and 1 *R. solanacearum* as *Ralstonia insidiosa*) compared to sequencing results. Both systems correctly identified all *Escherichia coli*, *Pseudomonas aeruginosa, Enterobacter* spp., *Proteus* spp.*, Serratia* spp., *Citrobacter* spp*.* and *Morganella* spp.

As shown in Table 2, Vitek 2 gave correct and acceptable identification for 255 (95.5%) of the 267 Gram-positive cocci, while it could not identify *Rhodococcus equi* as it is not present in its database. It misidentified 12 Gram-positive cocci: 1 *Staphylococcus epidermidis* misidentified as *Staphylococcus capitis*, 2 *Micrococcus luteus/lylae* as *Kocuria kristinae*, 1 *Streptococcus lutetiensis* as *Streptococcus infantarius*, 8 *Streptococcus mitis/oralis* as *Streptococcus constellatus*; 3, *Streptococcus peroris*; 1, *Streptococcus pluranimalium*; 1, *Streptococcus pentosaceus*; 1, *Streptococcus sanguis*; 1, *Streptococcus thoraltensis*; 1. VMS correctly identified at an acceptable level, 263 (98.5%) of the 267 Gram-positive cocci; *Rhodococcus equi* was not present in its database but identified by 16S rRNA sequencing. VMS misidentified 5 Gram-positive cocci: 1 *S. epidermidis* as *Staphylococcus capitis*, 1 *Staphylococcus warneri* as *Staphylococcus auricularis*, 1 *Staphylococcus lutetiensis* as *Staphylococcus infantarius,* 1 *S. mitis/oralis* as *S. constellatus*; and 1 *Streptococcus agalactiae* as *Nocardia asteroides*. Out of 267 Gram-positive cocci, BMS gave correct and acceptable identification for 237 (88.8%). It misidentified 30 Gram-positive cocci isolates. These were the following: 1 *Staphylococcus caprae* as *S. auricularis*, 1 *Staphylococcus warneri* as *S. epidermidis*, 2 *S. agalactiae* as *Alloiococcus otitis* and *Lactobacillus sakei*, 3 *Micrococcus luteus/lylae* as *Clostridium beijeriuckii* 1; and *Propionibacterium acnes* 2; 1 *Streptococcus parasanguis* as *Alloiococcus otitis*. Compared to sequencing results, 18 of 26 *S. mitis/oralis* group were misidentified as *Streptococcus pneumoniae.* In addition, 1 *S. gallolyticus* was misidentified as *P. acnes* and 1 *Streptococcus mutans* as *P. acnes*. All the 3 systems correctly identified all *Staphylococcus aureus*, *Streptococcus pyogenes, S. pneumoniae* and *Enterococcus* spp. to the genus and species levels.

Table 3 shows the discrepancies in the conventional and MALDI-TOF methods (Vitek 2, BMS and VMS) for identification of 16 Gram-negative cocci. Vitek 2 identified all Gram-negative cocci to the genus and species levels except 5 *Neisseria subflava* for which database is absent. VMS identified 15 (93.8%) of 16 Gram-negative cocci correctly, and misidentified 1 *Neisseria cinerea* as *N. subflava,* when compared to sequencing results. However, BMS correctly identified only 8 (50%) of 16 Gram-negative cocci to the species and genus levels. It correctly identified 2 of 8 *Haemophilus influenzae*, but with an ID score of <1.7 and it misidentified 1 *N. cinerea* as *Neisseria sicca* and 5 *N. subflava* as *Neisseria flava,* when compared to sequencing results.

The discrepancies and errors among Vitek 2, BMS and VMS for identification of 16 Gram-positive bacilli are shown in Table 4. Vitek 2 correctly identified only 4 of 16 Gram-positive bacilli. *Corynebacterium terpenotabidum, Corynebacterium striatum*, *Lactobacillus jensenii* and *Lactobacillus rhamnosus* are not present in Vitek 2 data base. Compared to sequencing results, Vitek 2 misidentified 1 *Nocardia otitidiscaviarum* as *Listeria monocytogenes*. VMS correctly identified to an acceptable level, 14 (87.5%) of 16 Gram-positive bacilli. It misidentified 1 *Corynebacterium terpenotabidum* as *Clostridium septicum* and 1 *N. otitidiscaviarum* as *L. monocytogenes.* BMS identified 13/16 (81.3%) Gram-positive bacilli, but misidentified *C. terpenotabidum* as *Clostridium beijerinckii*, 1 *Listeria ivanovii* as *L. monocytogenes* and 1 *N. otitidiscaviarum* as *L. monocytogenes*.

## Discussion and conclusions

MALDI-TOF MS systems have been now implemented in many clinical microbiology laboratories because of their efficiency, cost-effectiveness and rapid identification of different bacterial isolates. This technology is dependent on the reference strains included in their databases. Thus, when an isolate is tested, the species of the reference strain with the closest match is used for identification of the tested strain.

An important advantage of MALDI-TOF system is rapid identification of bacteria in just under 10 min compared to CPS that takes 16–20 h. The commercial systems allow the rapid identification of *S. aureus* but MALDI-TOF can recognize different coagulase negative staphylococci (CoNS). Identification of the exact species of CoNS may be very useful because some CoNS can cause serious infections and it may help to differentiate contaminants from true infections caused by CoNS [23]. A recent comparative study by Dupont *et al*., [24] in which MALDI–TOF MS and Vitek 2 were evaluated for the identification of 234 CoNS representing 20 different species showed that MALDI-TOF MS significantly performed better than Vitek 2 (93.2% versus 75.2%) with fewer misidentifications. In our study, all the 53 *S. aureus* isolates were correctly identified by MALDI-TOF MS and Vitek 2, while only 1 *S. epidermidis* was misidentified by Vitek 2 and 2 isolates each were misidentified by BMS and VMS.

*S. pneumoniae* is a major human pathogen and it has to be identified reliably and accurately in the clinical microbiology laboratory. In our hands, the CPS and both MALD-TOF systems identified it correctly which is discordant with the study of Seng *et al*. [5], which reported that about 50% of *S. pneumoniae* isolates were misidentified as *Streptococcus parasanguinis*, a member of *Streptococcus mitis* group. However, it should be pointed out that in our study BMS and VMS misidentified 18 of 26 and 1 of 26 *S. mitis/oralis* group as *S. pneumoniae*, respectively, a finding that may clinically impact patient management. Similar misidentification has been reported by others in which MALDI-TOF MS system was found to misidentify *S. mitis/oralis* as *S. pneumoniae* [6,25]. This misidentification may be explained by the fact that *S. pneumoniae* is strikingly similar to *S. mitis* genetically [26] and that the resolving power of MALDI-TOF is not sufficient to discriminate between the two species [18]. Therefore, supplementary tests such as optochin disk susceptibility and bile solubility are needed for the correct identification of *S. pneumoniae*. It is important to subject an isolate to these supplementary tests prior to reporting it as *S. pneumoniae* because insertion of additional spectra of these species into the BMS database may not solve the problem. In contrast to our findings, another study did not misidentify any of the 369 non-pneumococcal streptococci and related genera as *S. pneumoniae* [27]. Therefore, MALDI-TOF MS analysis cannot be used alone for identification of these organisms in all laboratories. It must be pointed out that all enterococci isolates were correctly identified to the species level by both MALDI-TOF MS systems which is concordant with the report by van Veen *et al*., [6].

An important similarity in the performance of both MALDI-TOF MS and Vitek 2 was their ability to correctly identify Gram-negative bacteria, particularly the *Enterobacteriaceae* with few exceptions to genus and species levels. Compared to the CPS identification of non-fermenting Gram-negative bacilli which is frequently protracted, expensive and difficult, these rapid systems appear to be superior. The explanation for the problems sometimes encountered with the CPS may be attributed to the mucoid nature of some of these organisms or the fact that they are inert biochemically [28]. Some previous studies reported misidentification of *S. maltophilia* as *Pseudomonas beteli*, and *Pseudomonas geniculata* or *Pseudomonas hibiscicola* by BMS. This is thought to be related to a high level of homology between the genera and the species [5,6]. However, all our 18 *S. maltophilia* were correctly identified by both BMS and VMS although 1 isolate which was identified by BMS had a low ID score of <1.7.

In this study, the *Corynebacterium* species collected in our routine practice were not identified by Vitek 2 primarily because of a lack of database for this species. Only 1 out of 6 isolates was misidentified as *Clostridium* species by both BMS and VMS. This may be explained by a lack of species diversity as previously alluded to by Dubois *et al*. [27]. In the past, Gram-positive bacilli were not routinely identified to species level as are Gram-negative rods isolated from clinical specimens. The use of MALDI-TOF MS in the clinical microbiology laboratory will provide species–level identification of these bacteria. Conceivably, identification of Gram-positive bacilli to species level may allow us to learn more about the real clinical significance of these organisms and perhaps help determine their hither - to underappreciated pathogenic potential.

An important limitation of this study was the relatively small number of Gram-negative cocci and Gram-positive bacilli encountered during this study which makes a general evaluation regarding their identification rather weak. A prospective evaluation study involving a large number of stock clinical isolates of these groups is underway.

Several studies have also assessed the use of MALDI-TOF MS to detect the mechanisms of antibiotic resistance in *Enterobacteriaceae*, such as identification of β-lactams and β-lactam modified/degraded products [29,30], detection of resistance proteins within the cell [31,32] and detection of mutations within resistance genes through mini-sequencing [33]. In addition, MALDI-TOF MS has been used in clinical practice to detect *Enterobacteriaceae* producing extended spectrum β-lactamase from positive blood cultures [34]. However, the limitation of these reports is that these mechanisms will detect only one type of resistance mechanism to certain antibiotics e.g. β-lactams, which makes the clinical implication of their findings unsure at this time. Another notable limitation of the rapid MALDI-TOF MS systems is that neither of them could perform susceptibility testing for all antibiotics. In effect, the physician still has to wait for another 24 h to make an informed and definitive decision on appropriate antibiotic therapy.

In conclusion, both MALDI-TOF MS systems have the potential to replace the conventional methods for identification of the majority of pathogens isolated in the clinical microbiology laboratory. MALDI-TOF is a rapid, simple, inexpensive, user-friendly and high-throughput proteomic technique for identification of clinically significant bacteria (except viridans streptococci) which can be implemented in a routine, conventional, laboratory setting.
